# Prognostic Value of Right Ventricular Dysfunction and Tricuspid Regurgitation in Patients with Severe Low-Flow Low-Gradient Aortic Stenosis

**DOI:** 10.1038/s41598-019-51166-0

**Published:** 2019-10-10

**Authors:** Robert Zilberszac, Andreas Gleiss, Ronny Schweitzer, Piergiorgio Bruno, Martin Andreas, Marlies Stelzmüller, Massimo Massetti, Wilfried Wisser, Günther Laufer, Thomas Binder, Harald Gabriel, Raphael Rosenhek

**Affiliations:** 1 Department of Cardiology, Medical University of Vienna, Vienna, Austria; 2 Center for Medical Statistics, Informatics, and Intelligent Systems, Section for Clinical Biometrics, Medical University of Vienna, Vienna, Austria; 3 Institute of Cardiology, Catholic University of Sacred Heart, Rome, Italy; 4 Department of Cardiac Surgery Medical University of Vienna, Vienna, Austria

**Keywords:** Valvular disease, Outcomes research

## Abstract

Long and mid-term data in Low-Flow Low-Gradient Aortic Stenosis (LFLG-AS) are scarce. The present study sought to identify predictors of outcome in a sizeable cohort of patients with LFLG-AS. 76 consecutive patients with LFLG-AS (defined by a mean gradient <40 mmHg, an aortic valve area ≤1 cm^2^ and an ejection fraction ≤50%) were prospectively enrolled and followed at regular intervals. Events defined as aortic valve replacement (AVR) and death were assessed and overall survival was determined. 44 patients underwent AVR (10 transcatheter and 34 surgical) whilst intervention was not performed in 32 patients, including 9 patients that died during a median waiting time of 4 months. Survival was significantly better after AVR with survival rates of 91.8% (CI 71.1–97.9%), 83.0% (CI 60.7–93.3%) and 56.3% (CI 32.1–74.8%) at 1,2 and 5 years as compared to 84.3% (CI 66.2–93.1%), 52.9% (CI 33.7–69.0%) and 30.3% (CI 14.6–47.5%), respectively, for patients managed conservatively (p = 0.017). The presence of right ventricular dysfunction (HR 3.47 [1.70–7.09]) and significant tricuspid regurgitation (TR) (HR 2.23 [1.13–4.39]) independently predicted overall mortality while the presence of significant TR (HR 3.40[1.38–8.35]) and higher aortic jet velocity (HR 0.91[0.82–1.00]) were independent predictors of mortality and survival after AVR. AVR is associated with improved long-term survival in patients with LFLG-AS. Treatment delays are associated with excessive mortality, warranting urgent treatment in eligible patients. Right ventricular involvement characterized by the presence of TR and/or right ventricular dysfunction, identifies patients at high risk of mortality under both conservative management and after AVR.

## Introduction

Calcific aortic stenosis (AS) is increasingly prevalent in an ageing population and the number of patients requiring treatment for AS is expected to rise further in the near future^1^. In 5–10% of patients with AS^2^, a “low-flow low-gradient AS” (LFLG-AS) situation, defined by an aortic valve area (AVA) ≤1 cm^2^ and a mean Gradient (mGrad) <40 mmHg in the presence of a left ventricular ejection fraction (LVEF) <50%^3,4^, is encountered. These patients have a worse prognosis than patients with “classical” high-gradient AS, irrespective of the treatment strategy^5,6^. Dobutamine stress echocardiography (DSE) is helpful^3,4^ to assess stenosis severity and LV flow reserve, which is predictive of outcome after SAVR^7^ but even patients without flow reserve may benefit from interventions^8^.

Severe symptoms, coronary artery disease (CAD), severe LV dysfunction; absence of LV flow reserve on DSE, B-type natriuretic peptides and myocardial fibrosis have been identified as predictors of adverse outcomes^9^. Since LFLG-AS constitutes an advanced disease stage, it is frequently associated with mitral regurgitation (MR), tricuspid regurgitation (TR) and RV dysfunction. Recent data indicate a potential predictive value of TR^10^ and right ventricular dysfunction (RVD)^11^.

The aim of the present study was to assess the predictive value of RVD, TR and MR on the long-term outcome of patients with LFLG-AS.

## Methods

### Study population

All consecutive patients that were seen in our heart valve clinic (HVC) between 1998 to 2014 who were found to have native aortic valve stenosis with mean gradient <40 mmHg, an aortic valve area [AVA] ≤1 cm^2^) and a reduced LVEF (≤50%) were included into the study when they had no additional hemodynamically significant rheumatic mitral stenosis or aortic regurgitation and had no history of previous cardiac surgery.

According to these criteria, 76 consecutive patients (28 female, median age 74 yrs) were identified.

The study was performed according to the Declaration of Helsinki and approved by the ethics committee of the Medical University of Vienna. In accordance with the ethics committee, written informed consent was not demanded due to the observational study design.

### Clinical data

As previously^12^, the following data were collected at baseline: age, gender, medications and medical history, history of hypercholesterolemia (cholesterol >200 mg/dL or patient undergoing lipid-lowering therapy at baseline), diabetes mellitus, arterial hypertension (use of antihypertensive medication or average blood pressure >140/90 mm Hg based on repeated measurements), CAD (history of myocardial infarction, angioplasty or angiographically documented coronary artery stenosis). The EuroSCORE II^13^ was calculated using baseline data.

### Echocardiography

As in our previous studies^12,14–18^, echocardiographic data were obtained by the use of commercially available ultrasound systems. All patients underwent a comprehensive examination including M-Mode, two-dimensional echocardiography, conventional and color Doppler conducted by an experienced echocardiographer. For the calculation of EF (by Simpson’s biplane formula), apical four-chamber and two-chamber views were used. Aortic jet velocities (AV-Vel) were measured by continuous-wave Doppler using multiple imaging windows including the apical three and five-chamber, right parasternal and suprasternal windows and taking care to record the highest velocity signal. Pressure gradients were estimated by the use of Bernoulli’s equation, and aortic valve area was calculated with the continuity equation. Stroke volume was measured in the LV outflow tract using pulsed-wave Doppler and indexed for BSA.

The degree of aortic valve calcification was scored according to criteria previously described^19^: 1- no calcification, 2- mild calcification (isolated, small spots), 3- moderate calcification (multiple bigger spots), 4- heavy calcification (extensive thickening/calcification of all cusps).

DSE was performed as described previously^20^. The protocol was started with intravenous administration of 2.5 µg/kg/minute dobutamine, with increments of 2.5 or 5 µg/kg/minute every 3 to 5 minutes to a maximum of 20 µg/kg/minute. At each step of the protocol, continuous-wave Doppler of the aortic valve velocity spectrum and pulsed-wave Doppler of the LV outflow tract velocity spectrum, was obtained. Presence of flow reserve was defined as >20% increase in stroke volume from rest to peak dobutamine stress. Patients with an increase in AV-Vel to ≥4 m/s or mean gradient to >30–40 mmHg in the presence of an AVA ≤1 cm^2^ at peak dobutamine dose were classified as having true-severe LFLG-AS^21^.

TR was quantified by an integrated approach that included valve morphology; RV, and right atrial volume load; inferior vena cava size; vena contracta width; proximal flow convergence radius and hepatic venous flow pattern. Moderate and severe TR were considered as significant TR^22–24^.

Right ventricular function was assessed by an integrative approach including visual assessment, tricuspid annular plane systolic excursion (TAPSE) in M-Mode, tissue doppler imaging (TDI) and right ventricular free-wall stain by speckle tracking when applicable, and classified as normal or reduced based on these criteria^25,26^.

### Heart valve clinic program and follow-up

As described previously^18^, patients were followed up prospectively after inclusion in our HVC follow-up program. Once the diagnosis of true-severe LFLG-AS was established, eligible patients were immediately referred to AVR and underwent a systematic preoperative work-up including coronary angiography, CT angiography of the aorta, carotid ultrasound and spirometry. After completion of the work-up, each case was discussed in a heart team consisting of cardiac surgeons as well as interventional and non-interventional cardiologists. Treatment decisions were individualized considering the patient’s risk profile, comorbidities, age and preferences. After valve interventions, cardiac rehabilitation programs (in both in – and outpatient settings) were available for all patients, and patients had a postinterventional follow-up visit in the HVC to assess the procedural outcome. Further follow-up exams in the HVC were scheduled at extended intervals, depending on individual procedural and clinical outcomes. Patients who refused to undergo screening for eventual valve interventions were encouraged to revise their decision and were scheduled for 6-monthly clinical and echocardiographic re-evaluation. For the assessment of survival and causes of death, the national mortality registry was queried and additional follow-up information was obtained from telephone interviews with the patients, their relatives and their physicians.

### Statistical analysis

Categorical baseline variables are described as counts and percentages and compared between groups using Chi-square tests. Continuous baseline variables are described as medians and quartiles and compared between groups using Wilcoxon rank-sum tests due to skewed distributions.

Survival rates (with 95% confidence intervals [CI]) at clinically relevant time points and median survival (with quartiles) are deduced from Kaplan-Meier estimates. In the case of time until valve intervention, rates and median are deduced from cumulative incidence functions accounting for death as a competing risk.

The potential influence of various prognostic factors on survival is investigated in a landmark analysis. For this purpose a landmark was set at 200 days after the index examination in the HVC to describe the survival outcome since the landmark (and the prognostic factors’ influence on survival) for all patients who already had a valve intervention up to 200 days versus those who had not (or not yet) had such an intervention. At 200 days, approximately 75% of the patients who ever had a valve intervention during their observation time already had their intervention. This analysis does not apply to patients who died or were censored before the landmark. For each prognostic factor an unadjusted hazard ratio (with 95% confidence interval) is estimated as well as a hazard ratio adjusted for baseline log EuroSCORE II. The models for both the unadjusted and the adjusted results, are stratified for valve intervention up to 200 days. In the tables we report uncorrected p-values and indicate statistical significance after multiplicity correction according to the method by Bonferroni-Holm using footnotes. In addition, the relative importance of each factor is quantified using the proportion of variation in the survival outcome explained by it^27^.

For postinterventional survival unadjusted results and results adjusted for baseline log EuroSCORE II as well as proportions of explained variation (PEVs) are reported in a similar way.

To investigate the potential effect of baseline characteristics on valve intervention, a Fine & Gray model with outcome time to valve intervention, taking into account death as a competing event, was used. Subdistribution hazard ratios (SHR) are provided with 95% confidence intervals (CI).

All analysis were performed using SAS 9.4 (SAS Institute Inc., 2016). P-values below 0.05 were considered to indicate statistical significance.

## Results

In total, 76 patients were included in the study from 1998 to 2014. The baseline clinical and echocardiographic characteristics of the patients as well as the pharmacological therapy are given in Table 1.

**Table 1:** Baseline patient characteristics.

	All Patients (n = 76)	No significant TR (n = 51)	Significant TR (n = 25)	p - Value
Gender (female), n (%)	28 (37%)	15 (29%)	13 (52%)	0.08
Age (years)	74 (64–81)	72 (69–74)	75 (71–79)	0.15
Peak aortic jet velocity (m/s)	3.4 (3.1–3.6)	3.3 (3.2–3.5)	3.3 (3.2–3.5)	0.76
Aortic mean gradient (mmHg)	27 (22–33)	28 (26–29)	26 (24–29)	0.29
Aortic valve area (cm^2^)	0.79 (0.68–0.90)	0.82 (0.78–0.87)	0.73 (0.66–0.80)	0.03
TAPSE (mm)	20 (14–24)	21 (18–23)	17 (13–20)	0.07
RV free-wall strain (%)	19 (15–26)	21 (17–26)	18 (12–24)	0.37
Left ventricular ejection fraction (%)	33 (29–38)	32 (29–36)	31 (23–38)	0.68
PA systolic pressure (mmHg)	54 (39–62)	48 (44–52)	59 (54–64)	0.0012
Interventricular septal thickness (cm)	13 (12–14)	13 (13–14)	13 (12–14)	0.27
Coronary artery disease, n (%)	44 (58%)	33 (64%)	11 (44%)	0.13
Hypertension, n (%)	47 (62%)	33 (64%)	14 (56%)	0.62
Diabetes mellitus, n (%)	32 (42%)	18 (35%)	14 (56%)	0.14
Hypercholesterolemia, n (%)	44 (58%)	32 (63%)	12 (48%)	0.32
COPD, n (%)	11 (14%)	9 (18%)	2 (8%)	0.32
Atrial fibrillation, n (%)	19 (25%)	9 (18%)	10 (40%)	0.05
Peripheral artery disease, n (%)	22 (29%)	14 (27%)	8 (32%)	0.79
Baseline EuroSCORE II* (%)	7.6 (3.5–12.9)	8.9 (6.3–11.5)	13.1 (9.3–16.9)	0.07
Beta blocker, n (%)	58 (77%)	38 (75%)	20 (83%)	0.56
ACE-Inhibitor/ARB, n (%)	59 (79%)	41 (80%)	18 (75%)	0.76
Statin, n (%)	45 (60%)	32 (63%)	13 (54%)	0.61
Acetylsalicylic acid, n (%)	31 (42%)	22 (44%)	9 (38%)	0.63
Thiazide diuretic, n (%)	19 (25%)	14 (27%)	5 (21%)	0.78
Loop diuretic, n (%)	48 (65%)	30 (59%)	18 (78%)	0.12
Aldosterone antagonist, n (%)	25 (33%)	14 (27%)	11 (46%)	0.13

n (%) for categorical and median (quartiles) for continuous variables. Abbrevations: COPD = Chronic Obstructive Pulmonary Disease; TAPSE = Tricuspid Annular Plane Systolic Excursion; PA = Pulmonary Artery; ACE-Inhibitor = Angiotensin Converting Enzyme Inhibitor; ARB = Angiotensine Receptor Blocker.

*Calculated retrospectively.

### Associated valve pathologies

By color Doppler, concomitant mild- to moderate aortic regurgitation was present in 53 patients. 59 patients had mild-to moderate MR and 16 patients had moderate-to-severe MR. While the mechanism of MR was secondary in all cases, additional degenerative changes were present in the majority of patients. 6 patients had additional degenerative calcification of the mitral apparatus with a mild-to-moderate stenotic effect. 51 pts had mild-to-moderate TR and moderate-to-severe TR was present in 15 patients. The median stroke volume was significantly lower in the presence of significant TR: 56.2 (quartiles 43.6–67.2) ml in patients without and 43.3 (37.1–54.8)) ml in patients with significant TR (p = 0.006), respectively. Also the stroke volume indexed to body surface area (BSA) was lower in patients with significant TR: 25.1 (20.6–28.1) ml/m^2^ vs. 28.6 (22.4–36.2) ml/m^2^ (p = 0.034). There was a trend for a lower stroke volume in the presence of significant MR: 46.1 (38.8–60.0) ml as compared to a stroke volume of 54.8 (41.1–67.2) ml for patients without significant MR (p = 0.06). However, the indexed stroke volume was comparable in patients with and without significant MR: 25.8 (20.6–28.6) ml/m^2^ vs. 29.3 (22.4–36.5) ml/m^2^, respectively (p = 0.10).

### Comorbidities

Medical comorbidities are given in Table 1. Almost 60% of the patients had CAD, 40% had diabetes and 30% had renal insufficiency or peripheral artery disease. At baseline, the EuroSCORE II for the entire patient population was 7.6 (quartiles 3.5–12.9).

### Symptomatic status

**Figure 1:**
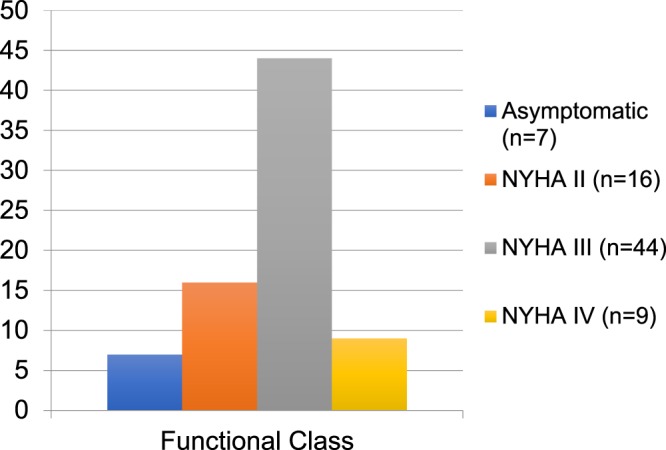
Symptomatic status at baseline. Asymptomatic patients (blue bar), patients in NYHA class II (red bar), patients in NYHA Class III (green bar) and patients in NYHA class IV (purple bar).

At baseline, 7 patients (9%) were asymptomatic, 16 patients (21%) were in NYHA or CCS Class II, 44 patients (58%) in NYHA/CCS Class III and 9 patients (12%) in NYHA/CCS Class IV. 5 patients (7%) also had an additional syncopal episode (Fig. 1).

### Valve replacement procedures

Valve replacement was performed in 44 patients (59.5% after 5 years) and included surgical aortic valve replacement (SAVR) with a biological prosthesis in 26 patients and with a mechanical prosthesis in 8 patients, transfemoral transcatheter aortic valve implantation (TAVI) in 8 patients and transapical TAVI in 2 patients. Valve interventions were performed after a median delay of 128 (lower quartile 81) days. Concomitant procedures included aortocoronary bypass surgery (16); tricuspid repair (3); mitral repair (4); and mitral replacement (3). Valve interventions were not performed in 32 patients for the following reasons: refusal by the patient (13); denial by the heart team (10); death on the waiting list (9). A 98-year old female patient underwent palliative balloon valvuloplasty for symptomatic relief after having refused both TAVI and SAVR. The connection between baseline characteristics and valve intervention was investigated: Only age (subdistribution hazard ratio (SHR) = 0.95 per year of life, CI: 0.93–0.98, p < 0.001) and log EuroSCORE II (SHR = 0.77 per doubling of score, CI: 0.64–0.93, p = 0.007) had a significant impact on time to valve intervention. Other clinical variables, including MR or TR severity and medical comorbidities had no significant effect.

### Peri- and postinterventional survival

27 of the patients that underwent valve interventions died during follow-up. There were three periinterventional deaths (within 30 days of interventions), all due to heart failure.

The reasons for the 24 late postprocedural deaths were: heart failure (13); myocardial infarction (1), liver failure (1), cancer (3), chronic obstructive pulmonary disease (COPD) (1), sudden death (1), kidney failure (1), sepsis (1); unknown (2). Postprocedural survival rates were 84.9% (CI 69.3–93.0%), 78.6% (CI 61.4–88.8%) and 45.1% (CI 26.6–62.0%) at 1, 2 and 5 years respectively.

### Predictors of postprocedural survival and mortality

**Figure 2:**
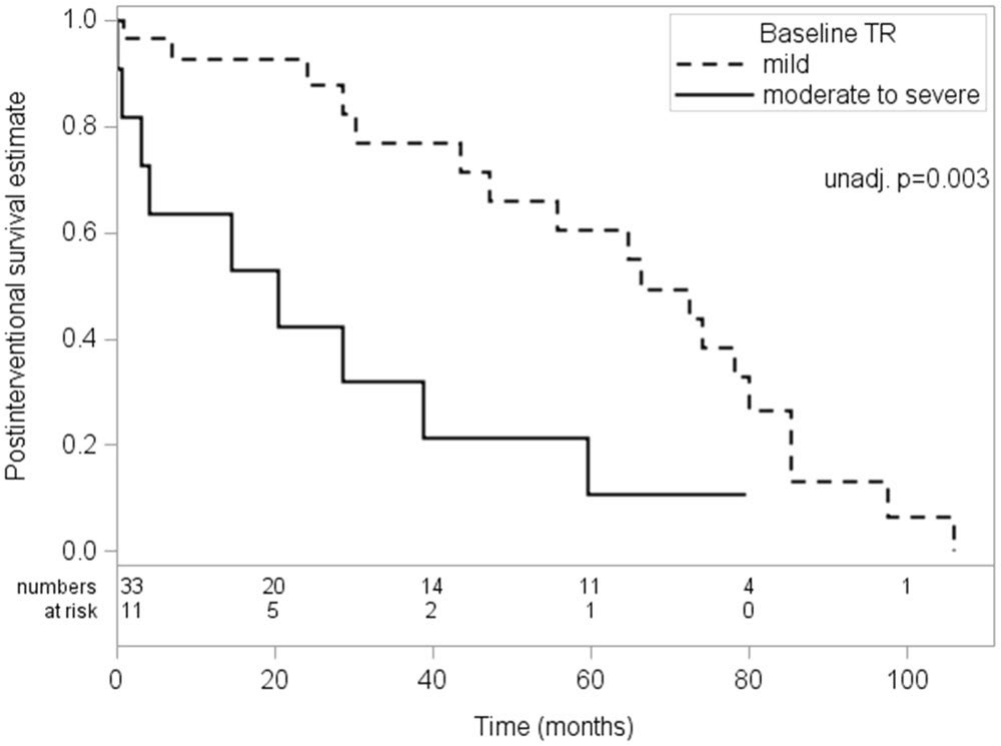
Postinterventional survival according to TR at baseline. Kaplan–Meier post-interventional survival estimates for patients with significant TR (solid line) vs. patients without significant TR (dashed line).

The predictors of postprocedural survival are presented in Table 2. The presence of at least moderate TR was the strongest predictor of postinterventional mortality (HR = 3.48, CI 1.38–8.35; p = 0.003) even when adjusting for the EuroSCORE II (HR = 3.40, CI 1.38–8.35; p = 0.008; PEV 10.7%). The respective postprocedural survival rates were 63.6% (CI 29.7–84.5%), 42.4% (CI 13.7–69.1%) and 10.6% (CI 0.0–37.3%) at 1,2 and 5 years for patients with significant TR as compared to 92.7% (CI 73.7–98.2%), 92.7% (CI 73.7–98.2%) and 60.4% (CI 35.5–78.2%) at 1,2 and 5 years for patients without significant TR (log-rank p = 0.003; Fig. 2).

**Table 2:** Postinterventional Survival: Analysis of Potential Clinical and Echocardiographic Predictors.

Parameter	Unadjusted HR (95% CI)	Unadjusted P - Value	Adjusted* HR (95% CI)	Adjusted* P - Value	Partial*** PEV %
Aortic regurgitation severity	0.71 (0.27–1.90)	0.499	0.86 (0.32–2.31)	0.767	0.0
Body mass index	1.02 (0.95–1.09)	0.660	1.03 (0.96–1.11)	0.414	0.0
Coronary artery disease	0.88 (0.37–2.09)	0.772	0.58 (0.23–1.48)	0.257	3.9
LV ejection fraction	1.04 (0.92–1.17)	0.562	1.03 (0.90–1.18)	0.644	0.0
Hypercholesterolemia	1.23 (0.54–2.80)	0.614	1.19 (0.52–2.71)	0.687	0.1
Hypertension	0.76 (0.34–1.72)	0.512	0.68 (0.30–1.55)	0.357	0.7
Aortic valve area**	1.07 (0.77–1.49)	0.676	1.21 (0.86–1.72)	0.273	1.5
Peak aortic jet velocity**	0.88 (0.81–0.96)	0.005	0.91 (0.82–1.00)	0.050	4.7
Atrial fibrillation vs. SR	1.14 (0.46–2.82)	0.774	1.09 (0.44–2.70)	0.846	1.2
PM vs. SR	1.59 (0.52–4.86)	0.419	1.61 (0.53–4.93)	0.406	
Left atrial diameter	1.02 (0.97–1.06)	0.446	1.02 (0.97–1.07)	0.520	2.7
Moderate-to-severe MR	1.06 (0.46–2.40)	0.898	1.39 (0.58–3.35)	0.461	2.2
Neurologic dysfunction	0.89 (0.12–6.72)	0.914	0.75 (0.10–5.66)	0.779	0.3
Peripheral artery disease	0.94 (0.41–2.17)	0.885	0.47 (0.18–1.20)	0.114	5.9
Right ventricular dysfunction	2.89 (1.22–6.84)	0.016	2.27 (0.88–5.87)	0.090	5.5
Moderate-to-severe TR	3.48 (1.47–8.27)	0.003	3.40 (1.38–8.35)	0.008	10.7
Diabetes	2.17 (0.96–4.94)	0.064	1.94 (0.84–4.46)	0.118	2.9

*Adjusted results from models adjusted for log of EuroSCORE II.

**Effects given for steps of 0.1 (effects of remaining continuous parameters for unit steps).

***Partial PEV values give proportion of variation explained in addition to log of Baseline euroSCORE II.

P-values are not corrected for testing multiple parameters.

In the unadjusted analysis, other factors associated with postprocedural survival and mortality were higher peak aortic jet velocities (HR = 0.28, CI 0.12–0.68; p = 0.005; PEV 6.6%) and RVD (HR = 2.89, CI 1.22–6.84); p = 0.016; PEV 7.8%).

### Overall survival

The probability of survival (including deaths during conservative management, periprocedural and late postprocedural deaths) was 73.2% (CI 61.2–82.0%), 55.6% (CI 42.8–66.6%) and 34.8% (CI 22.8–47.0%) at 1, 2 and 5 years respectively.

In addition to the 27 deaths in patients who underwent valve interventions, all but two of the 32 patients that were managed conservatively died during follow-up. The reasons were malignant disease (3), myocardial infarction (2), COPD (1), periprocedural electromechanic dissociation during palliative balloon valvuloplasty (1), Clostridium difficile colitis (1), stroke (1) and heart failure (23).

### Predictors of overall survival and mortality

**Figure 3:**
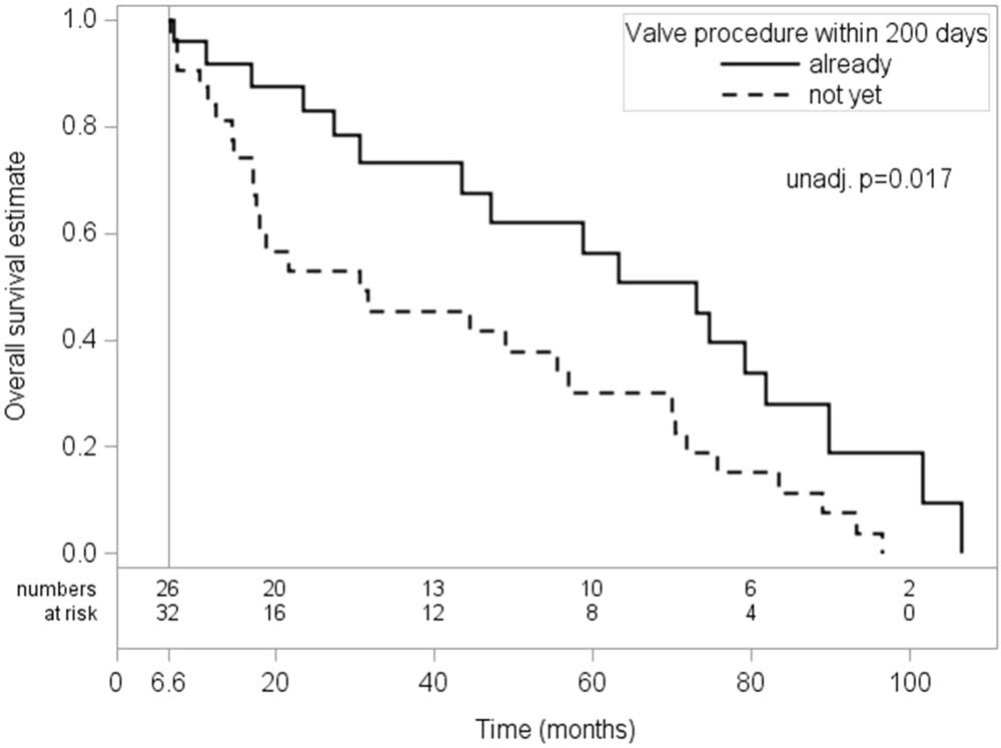
Overall survival according to treatment strategy. Kaplan–Meier overall survival estimates for patients still under study at 200 days (landmark analysis) who underwent valve interventions within 200 days (solid line) vs. patients who had not (yet) undergone valve intervention up to 200 days (dashed line).

According to a landmark analysis with a landmark at 200 days after baseline, valve interventions yielded a survival benefit with respective overall survival rates of 91.8% (CI 71.1–97.9%), 83.0% (CI 60.7–93.3%) and 56.3% (CI 32.1–74.8%) at 1,2 and 5 years after baseline for patients having undergone valve interventions within 200 days as compared to 84.3% (CI 66.2–93.1%), 52.9% (CI 33.7–69.0%) and 30.3% (CI 14.6–47.5%) at 1,2 and 5 years for patients who had not (yet) undergone valve interventions (log-rank p = 0.017, HR adjusted for EuroSCORE II = 0.53, p = 0.053, Fig. 3).

**Figure 4:**
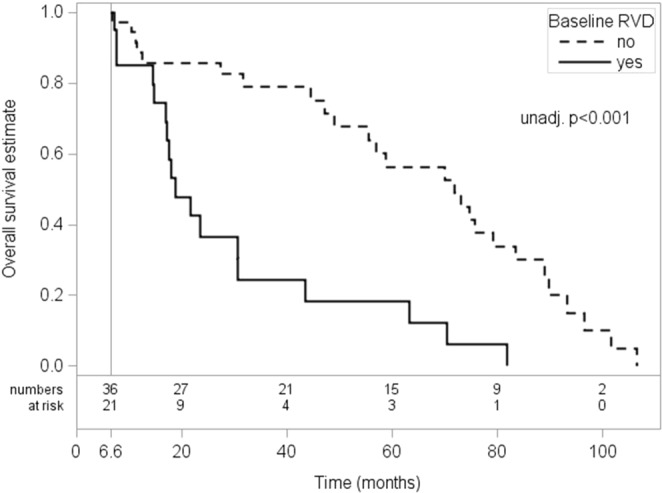
Overall survival according to RVD at baseline. Kaplan–Meier overall survival estimates for patients still under study at 200 days (landmark analysis) with RVD (solid line) vs. patients without RVD (dashed line).

The predictors of overall survival are presented in Table 3. RVD was the strongest predictor of overall mortality, even when adjusting for valve interventions. Survival rates for patients with RVD were 64.4% (CI 43.8–79.0%), 27.6% (CI 12.1–45.6%) and 13.8% (CI 3.6–30.7%) at 1,2 and 5 years for patients with RVD as compared to 78.6% (CI 62.8–88.3%), 76.0% (CI 60.0–86.4%) and 50.0% (CI 32.2–65.4%) at 1,2 and 5 years for patients without RVD (p < 0.001; Fig. 4). The presence of CAD was associated with lesser overall mortality (HR = 0.30, p = 0.004, PEV = 7.6%).

**Table 3:** Overall Survival from day 200: Landmark Analysis of Potential Clinical and Echocardiographic Predictors.

Parameter	Unadjusted HR (95% CI)	Unadjusted P - Value	Adjusted* HR (95% CI)	Adjusted* P - Value	Partial*** PEV %
Aortic regurgitation severity	0.59 (0.25–1.39)	0.225	0.79 (0.32–1.92)	0.596	2.1
Body mass index	0.99 (0.94–1.05	0.764	0.99 (0.93–1.05)	0.777	0.0
Coronary artery disease	0.62 (0.32–1.19)	0.148	0.30 (0.14–0.68)	0.004	7.6
LV ejection fraction	0.95 (0.88–1.03)	0.198	0.97 (0.89–1.05)	0.446	0.8
Hypercholesterolemia	0.86 (0.43–1.70)	0.656	0.62 (0.29–1.33)	0.218	0.4
Hypertension	0.70 (0.37–1.35)	0.287	0.67 (0.35–1.29)	0.230	0.4
Aortic valve area**	0.91 (0.72–1.16)	0.491	0.98 (0.76–1.28)	0.938	0.0
Peak aortic-jet velocity**	0.95 (0.89–1.01)	0.075	0.96 (0.91–1.03)	0.215	1.8
Atrial fibrillation vs. SR	1.34 (0.64–2.78)	0.436	1.44 (0.69–3.00)	0.334	0.7
PM vs. SR	1.36 (0.50–3.69)	0.544	1.46 (0.53–4.97)	0.464	
Left atrial diameter	1.03 (0.99–1.07)	0.121	1.03 (0.99–1.07)	0.199	2.1
Moderate-to-severe MR	1.10 (0.58–2.07)	0.778	1.13 (0.60–2.13)	0.710	0.7
Neurologic dysfunction	2.15 (0.62–7.44)	0.229	2.00 (0.58–6.90)	0.275	0.8
Peripheral artery disease	1.33 (0.70–2.55)	0.387	0.96 (0.47–1.96)	0.903	1.0
Right ventricular dysfunction	3.85 (1.92–7.70)	<0.001*	3.47 (1.70–7.09)	<0.001	11.9
Moderate-to-severe TR	2.53 (1.31–4.88)	0.006	2.23 (1.13–4.39)	0.021	5.6
Diabetes	1.41 (0.75–2.64)	0.280	1.31 (0.70–2.47)	0.395	0.8

*Adjusted results from models adjusted for log of EuroSCORE II and stratified for valve intervention up to the landmark.

**Effects given for steps of 0.1 (effects of remaining continuous parameters for unit steps).

***Partial PEV values give proportion of variation explained in addition to log of Baseline euroSCORE II.

All results from models stratified for valve interventions up to landmark (200 days); adjusted results from models adjusted for log of Baseline euroSCORE II.

P-values are not corrected for testing multiple parameters, significance after Bonferroni-Holm correction is indicated by bold face.

## Discussion

Patients with severe LFLG-AS are often elderly, frail and multimorbid and their management is based on limited evidence^9^. This is an observational study, aiming to investigate outcomes and to define potential prognostic factors for this specific subgroup of patients. In particular, we were able to demonstrate that well-selected patients benefit from aortic valve interventions, while interventions might be futile in patients that exhibit specific markers of very high risk, among them those with right ventricular affection, i.e RVD or significant TR.

### Prognostic impact of aortic valve replacement

Although aortic valve intervention improves both symptoms and survival, the overall morbidity and mortality have been reported to be high in the setting of LFLG AS, highlighting the importance of both risk assessment to identify patients in whom invasive treatment is most appropriate, and of state of the art care, during both hospitalization and postinterventional cardiac rehabilitation^28^. Despite a high-risk setting encountered in most of the included patients, highlighted by a median baseline EuroSCORE II of 7.6% and a median age of 74 years, the periprocedural mortality rate of 7% was low in comparison to reported perioperative mortality rates of 16%^29^ and 22%^30^ in earlier series. TAVI might be of particular benefit in these high-risk patients with recent multicentre registry data reporting a 3.8% 30-day mortality^8^.

While the present study confirms a sustainable survival benefit associated with aortic valve replacement, it also highlights a non-negligible long-term mortality of 55% (due to cardiac causes in 63% of cases) at 5 years among patients having undergone successful valve replacement. Careful patient selection, and individualized treatment strategies, planned in interdisciplinary heart teams are thus paramount in these patients.

### Importance of urgent treatment

Mortality was very high among patients on the waiting list for valve interventions: during a median delay of 4 months from the date first seen in the HVC until AVR/TAVI the mortality rate was 17%, principally due to cardiac causes (63%). Patients with LFLG-AS thus have a higher mortality on the waiting list than patients with symptomatic severe AS and a normal EF^31,32^. Treatment delays thus expose patients to an unacceptable risk and strategies should be implemented to avoid them once a patient has been deemed eligible for invasive treatment options. Several factors might contribute to those deferments. Elderly patients might be reluctant to undergo invasive procedures, partly due to a lacking perception of symptoms and the frequent request for time to contemplate their decision. We previously demonstrated that serial follow-up visits in HVCs might encourage patients who initially refused a valve intervention to reconsider their decision^18^.

In addition, the necessity to perform additional exams for refinement of the diagnosis such as DSE might contribute to further treatment delays. Interestingly, the importance of DSE has recently been challenged: in a multicentre registry of 287 patients^8^, DSE was of no predictive value with regards to recovery of LVEF or adverse events after TAVI. In fact, intervention should now be considered even in the absence of contractile reserve, given the positive impact on long-term survival^30^ and the potential for recovery of LV function^33^.

### Predictive value of right ventricular dysfunction and tricuspid regurgitation

Elevated filling pressures that are found in patients with long-standing disease may lead to pulmonary vascular remodelling, pulmonary hypertension and finally, RVD^34^.

In the present series, right-sided involvement, i.e. significant TR or RVD, was associated with a particularly high mortality, regardless of the treatment strategy.

RVD was the strongest independent predictor of overall mortality, even after adjustment for the baseline EuroSCORE II, which, among others incorporates LV function, age, mobility and major comorbidity^13^. Even after adjustment for valve intervention, RVD and TR were still predictive of excess mortality, indicating that the benefits of AVR might be mitigated in the presence of right-sided involvement.

The lower mortality that was observed in patients with CAD might, in our opinion, be explained by a contributory effect of myocardial revascularization on recovery by recruiting viable but hibernating areas^35^.

Postprocedural mortality was strongly associated with the preprocedural presence of significant TR and to a lesser extent by RVD. This might be explained by the fact that only very few patients with overt right ventricular dysfunction were eventually referred to AVR. On the other hand, the presence of TR (which is frequently associated with right ventricular dysfunction^36^) might conceal early stages of right ventricular failure^37^.

This is further substantiated by the association of significant TR but not of MR with lower stroke volumes, highlighting that right-sided involvement indicates more advanced disease^23^.

Dahou *et al*.^10^ describe a significantly higher 30-day mortality in patients with LFLG-AS who underwent AVR when significant TR was present. However, AVR was nonetheless associated with a survival benefit, even after adjustment for RV dysfunction or TR. The poor outcome of patients with right ventricular dysfunction and TR has previously been demonstrated in the settings of chronic heart failure^22,37^, in patients who underwent TAVI^38^, as well as in a small subset of patients with LFLG-AS^10^. Moderate-to-severe TR is also associated with dismal outcome in patients with severe AS and concomitant MR^16^.

Speckle tracking may identify reduced RV free wall strain, which is associated with increased mortality in patients with LFLG-AS^11^. However, RV free wall strain is limited by its dependence on RV loading conditions as well as RV size and shape and the fact that, depending on the region of interest chosen, erroneous measurement of pericardial or atrial structures might occur^25^. Furthermore, the software that is used to measure RV strain has been designed for LV measurements.

### Study limitations

While the sample size may seem relatively small, the present study compares well to the size of previous series of patients with LFLG-AS. Between 1990 and 2015, Levy *et al*.^29^ had included 217 patients in 11 centers, corresponding to 20 patients per center or 1.3 patients per center and year. In agreement with this multicentre registry, the majority of the patients in the present study had contractile reserve.

Speckle tracking and tissue Doppler have only been introduced recently and therefore were only available in a limited number of patients.

Only a limited number of the patients in the present study underwent invasive valve procedures, in particular TAVI (either based on heart team decisions favoring either conservative or surgical treatment, or due to inclusion in the pre-TAVI era).

Multidetector computed tomography to quantify aortic valve calcification^39^ was not systematically used in the present study, since the established strategy was to evaluate AS severity by DSE.

The use of EuroSCORE is intended for operative risk stratification only but has nevertheless been demonstrated to be useful to assess risk in patients managed conservatively^40^. Adjusting for EuroSCORE does not necessarily imply adjustment for all individual variables included in the score.

## Conclusion

Aortic valve replacement leads to improved long-term survival in patients with LFLG-AS. Treatment delays are associated with excessive mortality, warranting urgent treatment in eligible patients. Right ventricular involvement characterized by the presence of tricuspid regurgitation and/or right ventricular dysfunction, identifies patients at high risk of mortality under both conservative management and after valve replacement.

## References

[1] Badheka AO (2015). Trends of Hospitalizations in the United States from 2000 to 2012 of Patients >60 Years With Aortic Valve Disease. Am J Cardiol.

[2] Iung B (2003). A prospective survey of patients with valvular heart disease in Europe: The Euro Heart Survey on Valvular Heart Disease. Eur. Heart J..

[3] Vahanian A (2012). Guidelines on the management of valvular heart disease (version 2012): the Joint Task Force on the Management of Valvular Heart Disease of the European Society of Cardiology (ESC) and the European Association for Cardio-Thoracic Surgery (EACTS). Eur. J. Cardiothorac. Surg..

[4] Nishimura RA (2014). AHA/ACC Guideline for the Management of Patients With Valvular Heart Disease: A Report of the American College of Cardiology/American Heart Association Task Force on Practice Guidelines. J. Am. Coll. Cardiol..

[5] Connolly HM (1997). Aortic valve replacement for aortic stenosis with severe left ventricular dysfunction. Prognostic indicators. Circulation.

[6] Clavel MA (2015). Impact of classic and paradoxical low flow on survival after aortic valve replacement for severe aortic stenosis. J. Am. Coll. Cardiol..

[7] Monin JL (2003). Low-gradient aortic stenosis: operative risk stratification and predictors for long-term outcome: a multicenter study using dobutamine stress hemodynamics. Circulation.

[8] Ribeiro HB (2018). Transcatheter Aortic Valve Replacement in Patients With Low-Flow, Low-Gradient Aortic Stenosis: The TOPAS-TAVI Registry. J. Am. Coll. Cardiol..

[9] Clavel MA, Magne J, Pibarot P (2016). Low-gradient aortic stenosis. Eur. Heart J..

[10] Dahou A (2015). Tricuspid Regurgitation Is Associated With Increased Risk of Mortality in Patients With Low-Flow Low-Gradient Aortic Stenosis and Reduced Ejection Fraction: Results of the Multicenter TOPAS Study (True or Pseudo-Severe Aortic Stenosis). JACC Cardiovasc. Interv..

[11] Dahou A (2016). Right ventricular longitudinal strain for risk stratification in low-flow, low-gradient aortic stenosis with low ejection fraction. Heart.

[12] Zilberszac R (2017). Asymptomatic Severe Aortic Stenosis in the Elderly. JACC Cardiovasc Imaging.

[13] Nashef, S. A. *et al*. EuroSCORE II. *Eur. J. Cardiothorac. Surg*. **41**, 734–744; discussion 744–735, 10.1093/ejcts/ezs043 (2012). 10.1093/ejcts/ezs043 22378855

[14] Rosenhek R (2010). Natural history of very severe aortic stenosis. Circulation.

[15] Zilberszac R (2013). Outcome of combined stenotic and regurgitant aortic valve disease. J Am Coll Cardiol.

[16] Zilberszac R (2018). Prognostic relevance of mitral and tricuspid regurgitation in patients with severe aortic stenosis. Eur Heart J Cardiovasc Imaging.

[17] Zilberszac R (2018). Long-Term Outcome of Active Surveillance in Severe But Asymptomatic Primary Mitral Regurgitation. JACC Cardiovasc Imaging.

[18] Zilberszac R (2017). Role of a heart valve clinic programme in the management of patients with aortic stenosis. Eur Heart J Cardiovasc Imaging.

[19] Rosenhek R (2000). Predictors of outcome in severe, asymptomatic aortic stenosis. N Engl J Med.

[20] deFilippi CR (1995). Usefulness of dobutamine echocardiography in distinguishing severe from nonsevere valvular aortic stenosis in patients with depressed left ventricular function and low transvalvular gradients. Am. J. Cardiol..

[21] Baumgartner HC (2017). Recommendations on the echocardiographic assessment of aortic valve stenosis: a focused update from the European Association of Cardiovascular Imaging and the American Society of Echocardiography. Eur. Heart J. Cardiovasc. Imaging.

[22] Neuhold S (2013). Impact of tricuspid regurgitation on survival in patients with chronic heart failure: unexpected findings of a long-term observational study. Eur. Heart J..

[23] Taramasso M (2012). The growing clinical importance of secondary tricuspid regurgitation. J. Am. Coll. Cardiol..

[24] Kammerlander AA (2014). Right ventricular dysfunction, but not tricuspid regurgitation, is associated with outcome late after left heart valve procedure. J. Am. Coll. Cardiol..

[25] Lang RM (2015). Recommendations for cardiac chamber quantification by echocardiography in adults: an update from the American Society of Echocardiography and the European Association of Cardiovascular Imaging. Eur. Heart J. Cardiovasc. Imaging.

[26] Rudski, L. G. *et al*. Guidelines for the echocardiographic assessment of the right heart in adults: a report from the American Society of Echocardiography endorsed by the European Association of Echocardiography, a registered branch of the European Society of Cardiology, and the Canadian Society of Echocardiography. *J Am Soc Echocardiogr* **23**, 685–713; quiz 786–688, 10.1016/j.echo.2010.05.010 (2010). 10.1016/j.echo.2010.05.010 20620859

[27] Gleiss A (2016). Statistical controversies in clinical research: the importance of importance. Ann Oncol.

[28] Sibilitz KL (2016). Exercise-based cardiac rehabilitation for adults after heart valve surgery. Cochrane Database Syst Rev.

[29] Levy F (2008). Aortic valve replacement for low-flow/low-gradient aortic stenosis operative risk stratification and long-term outcome: a European multicenter study. J. Am. Coll. Cardiol..

[30] Tribouilloy C (2009). Outcome after aortic valve replacement for low-flow/low-gradient aortic stenosis without contractile reserve on dobutamine stress echocardiography. J. Am. Coll. Cardiol..

[31] Lund O (1996). Mortality and worsening of prognostic profile during waiting time for valve replacement in aortic stenosis. Thorac. Cardiovasc. Surg..

[32] Munt BI, Humphries KH, Gao M, Moss RR, Thompson CR (2006). True versus reported waiting times for valvular aortic stenosis surgery. Can. J. Cardiol..

[33] Quere JP (2006). Influence of preoperative left ventricular contractile reserve on postoperative ejection fraction in low-gradient aortic stenosis. Circulation.

[34] Cavalcante JL (2016). Right Ventricular Function and Prognosis in Patients with Low-Flow, Low-Gradient Severe Aortic Stenosis. J. Am. Soc. Echocardiogr..

[35] Orlandini A (2015). Myocardial viability for decision-making concerning revascularization in patients with left ventricular dysfunction and coronary artery disease: a meta-analysis of non-randomized and randomized studies. Int J Cardiol.

[36] Vargas Abello LM (2013). Understanding right ventricular dysfunction and functional tricuspid regurgitation accompanying mitral valve disease. J. Thorac. Cardiovasc. Surg..

[37] Nath J, Foster E, Heidenreich PA (2004). Impact of tricuspid regurgitation on long-term survival. J. Am. Coll. Cardiol..

[38] Asami, M. *et al*. Prognostic Value of Right Ventricular Dysfunction on Clinical Outcomes After Transcatheter Aortic Valve Replacement. *JACC Cardiovasc. Imaging*, 10.1016/j.jcmg.2017.12.015 (2018). 10.1016/j.jcmg.2017.12.015 29454762

[39] Clavel MA (2014). Impact of aortic valve calcification, as measured by MDCT, on survival in patients with aortic stenosis: results of an international registry study. J. Am. Coll. Cardiol..

[40] Bernal E (2017). Conservative management in very elderly patients with severe aortic stenosis: Time to change?. J. Cardiol..

